# Clinical application of long-read sequencing in newborn genetic screening for congenital adrenal hyperplasia

**DOI:** 10.3389/fgene.2026.1894466

**Published:** 2026-08-10

**Authors:** Peiran Zhao, Xiaolong Qiu, Qingying Lin, Ting Huang, Yinglin Zeng, Jinfu Zhou, Liangpu Xu

**Affiliations:** Medical Genetic Diagnosis and Therapy Center, Fujian Key Laboratory for Prenatal Diagnosis and Birth Defect, Fujian Maternity and Child Health Hospital College of Clinical Medicine for Obstetrics and Gynecology and Pediatrics, Fujian Medical University, Fuzhou, Fujian, China

**Keywords:** 17α-hydroxyprogesterone, congenital adrenal hyperplasia, CYP21A2, genetic variation, long-read sequencing, newborn screening

## Abstract

**Background:**

Screening for congenital adrenal hyperplasia (CAH) relying solely on 17α-hydroxyprogesterone (17α-OHP) presents limited diagnostic performance, highlighting an urgent need to develop more robust screening strategies for neonates.

**Methods:**

We conducted retrospective and prospective cohort studies to explore the clinical applicability of long-read sequencing (LRS) in CAH genetic testing within primary and secondary newborn screening (NBS) systems, respectively. The retrospective cohort comprised 100,145 neonates who underwent routine 17α-OHP primary CAH screening at the Fujian Provincial Newborn Screening Center from January 1 to December 31, 2019. Among these infants, 52 full-term screen-positive neonates received further LRS-based CAH genotyping. The prospective cohort enrolled 2,100 newborns recruited from Fujian Maternity and Child Health Hospital between May 1 and May 31, 2023, who underwent simultaneous 17α-OHP measurement and LRS-mediated CAH genetic analysis.

**Results:**

In the retrospective cohort, the positive rate of 17α-OHP screening was 0.19% (190/100,145, 95% CI: 0.17%–0.21%), and five infants were definitively diagnosed with CAH, corresponding to a disease prevalence of 1:20,029. LRS genotyping successfully identified five neonates harboring pathogenic *CYP21A2* mutations consistent with confirmed genetic diagnosis. In this prospective cohort study, two newborns (1/1050) with normal 17α-OHP concentrations were found to carry biallelic pathogenic variants in the *CYP21A2* gene. In addition, 88 neonates (4.2%) with normal 17α-OHP levels were found to carry heterozygous CAH-related variants. Among these heterozygotes, 85 individuals harbored *CYP21A2* variants, representing 32 distinct genotypes. The calculated carrier frequencies were 1 in 78 for classic CAH and 1 in 40 for non-classic CAH.

**Conclusion:**

LRS-integrated genetic newborn screening exhibits favorable efficacy for CAH identification. This combined screening modality holds great promise for wide implementation in routine neonatal screening practice.

## 1 Introduction

Congenital adrenal hyperplasia (CAH, OMIM #201910) is a group of autosomal recessive genetic disorders characterized by deficiencies in multiple enzymes involved in the adrenal steroidogenic pathway, leading to a variety of complex hormonal imbalances (Auer et al., 2023). CAH exhibits high clinical and genetic heterogeneity, which severely impairs patients’ quality of life. Clinically, more than 95% of CAH cases are caused by 21-hydroxylase deficiency (21-OHD), which disrupts the synthesis of cortisol, aldosterone, and androgens. Based on the severity of enzymatic deficiency, 21-OHD can be categorized into three subtypes: classic salt-wasting (SW), classic simple virilizing (SV), and non-classic congenital adrenal hyperplasia (NCCAH) (Merke and Auchus, 2020; Auer et al., 2023). The SW subtype, resulting from deficiencies in both cortisol and aldosterone, presents the most severe phenotype, including hypoglycemia and acute adrenal crisis; if left untreated, it can be fatal in the neonatal period. The SV subtype largely preserves mineralocorticoid synthesis, leading to a less severe clinical presentation; however, under conditions of major physical stress (e.g., trauma and surgery), the risk of salt loss is approximately 10%.The NCCAH subtype retains 20%–80% of *in vitro* enzyme activity, which may not compromise cortisol synthesis but can cause variable overproduction of adrenal androgens. In addition to 21-OHD, other rare forms of CAH exist: variants in the *CYP11B1*, *CYP17A1*, *HSD3B2*, and *StAR* genes, which are involved in steroid biosynthesis, can also lead to CAH (Miller, 2018). In China, the incidence of classic CAH is approximately 1 in 23,024 live births, while the incidence of non-classic CAH is considerably higher, at around 1 in 1,000 live births (Falhammar and Nordenström, 2015; Miller, 2018). The carrier frequency of classic CAH alleles in the general population is approximately 1 in 60 (Speiser et al., 2018).

Newborn screening (NBS) is a successful and comprehensive public health initiative that enables the effective identification of infants at high risk of rare childhood-onset disorders and the timely administration of therapeutic interventions prior to disease manifestation. NBS for CAH, which detects elevated 17α-hydroxyprogesterone (17α-OHP) concentrations via fluoroimmunoassay, can effectively identify the severe, life-threatening SW and SV subtypes. However, it fails to detect the less severe SV and non-classic (NC) subtypes due to their obscure phenotypes and marginally increased 17α-OHP levels (Hayashi et al., 2017). Furthermore, 17α-OHP concentrations are easily influenced by birth weight and gestational age at delivery, resulting in a false positive rate as high as 9.4% (Hayashi et al., 2017). Therefore, the development of effective screening techniques is particularly crucial.

With the rapid development of next-generation sequencing (NGS) technology and its application in the molecular diagnosis of genetic diseases, new opportunities have been provided for DNA-based neonatal genetic screening. The *CYP21A2* gene, which encodes 21-hydroxylase, is located at the 6p21.3 locus on the short arm of chromosome 6 and consists of 10 exons. Its non-functional pseudogene, *CYP21A1P*, is tandemly arranged with *CYP21A2*, with an approximate distance of 30 kilobases (kb) between the two (Witchel, 2017). These two genes share a high degree of nucleotide sequence homology, with 98% homology in exonic regions and 96% homology in intronic regions (Carvalho et al., 2021). In addition, *CYP21A2* and *CYP21A1P*, together with their adjacent genes, constitute the bimodular RCCX haplotype (RP1-C4A-*CYP21A1P*-TNXA-RP2-C4B-*CYP21A2*-TNXB) (Saraf et al., 2024). The high homology and tandem repeat sequences between functional genes (RP1, *CYP21A2*, and TNXB) and their corresponding pseudogenes (RP2, *CYP21A1P*, and TNXA) significantly increase the incidence of gene conversion, deletion, duplication, and non-functional chimeric gene formation during meiosis (El-Maouche et al., 2017). Consequently, approximately 75% of *CYP21A2* variants—particularly single nucleotide variations (SNVs) and small insertions and deletions (indels)—originate from the non-functional pseudogene via microconversion. The remaining 25% are large-fragment deletions/duplications and chimeric genes (*CYP21A1P*/*CYP21A2* chimeras or TNXA/TNXB chimeras (Chen et al., 2012). However, current NGS-based genotyping assays are not suitable for *CYP21A2* genotyping due to their short read lengths and relatively low analytical sensitivity in highly homologous sequences and intergenic recombination events.

Long-read sequencing (LRS), also referred to as third-generation sequencing technology, is a novel nucleotide sequencing approach that can directly detect full-length genes and their flanking regions, as well as discriminate between highly homologous genes. Furthermore, LRS is capable of identifying the cis-trans relationships of multiple variants and detecting deletions/duplications and large-fragment conversions. Recent studies have demonstrated that LRS is a powerful tool for the accurate genetic diagnosis of CAH (Liu et al., 2022; Li et al., 2023). In the present study, LRS-based CAH genotyping was integrated into the NBS program to systematically evaluate its clinical application value in neonatal CAH screening.

## 2 Methods

### 2.1 Study design

This study was conducted to assess the clinical application value of LRS for CAH gene detection in first-tier and second-tier NBS for CAH, utilizing a population-based retrospective cohort study and a prospective cohort study, respectively. The retrospective cohort included newborns recruited from the Fujian Provincial Newborn Screening Center, China, between January 1 and December 31, 2019, where neonatal 17α-OHP concentrations were measured for first-tier CAH NBS. Infants with positive 17α-OHP screening results underwent second-tier NBS using LRS for CAH gene detection. The prospective cohort included newborns enrolled from Fujian Maternity and Child Health Hospital between May 1 and May 31, 2023. Neonatal 17α-OHP concentration measurement and LRS-based CAH genotyping were performed simultaneously as first-tier NBS for CAH. Meanwhile, the genotypes of CAH patients were analyzed using multiplex ligation-dependent probe amplification (MLPA) and Sanger sequencing. This study was conducted in compliance with the Declaration of Helsinki and approved by the Ethics Review Committee of Fujian Provincial Maternity and Child Health Hospital (approval number: 2023KY017). Written informed consent was obtained from the parents or guardians of all participants after a detailed explanation of the study objectives.

### 2.2 NBS for 17α-OHP level

The workflow of NBS was performed according to the previously described procedure. Briefly, dried blood spot samples were collected within 3–7 days after birth and transported to the NBS center via a cold-chain transportation system. 17α-OHP concentrations were detected using the GSP Neonatal 17α-OHP Progesterone Kit (PerkinElmer, Turku, Finland) with the time-resolved fluorescence method, strictly following the manufacturer’s instructions. A threshold value of 17α-OHP concentration >12 nmol/L was defined as positive for CAH screening in full-term neonates (gestational age at delivery ≥ 37 weeks and birth weight ≥ 2,500 g), while a threshold of >25 nmol/L was set for premature neonates (gestational age at delivery < 37 weeks) or those with low birth weight (<2,500 g). Neonates with positive screening results were recalled, and an additional dried blood spot sample was collected for re-evaluation using the same GSP Neonatal 17α-OHP Progesterone Kit.

### 2.3 Long-read sequencing and data analysis

Heel-prick dried blood spot (DBS) samples were collected from newborns at postnatal days 3–7, followed by cold-chain delivery to the central molecular laboratory within 48 h. A sequential laboratory workflow was implemented, including genomic DNA extraction from DBS punches, long-range PCR amplification of CAH-associated genes, SMRT library preparation, sequencing on the PacBio Sequel II platform, circular consensus sequencing (CCS) read filtering, variant calling, variant classification in accordance with ACMG criteria, and final clinical report generation. The end-to-end laboratory turnaround time (TAT), defined as the interval between DBS receipt by the laboratory and release of the finalized genetic report, was 2 working days. When accounting for the full clinical screening pipeline under routine laboratory throughput, the overall comprehensive TAT reached 7 calendar days.

Genomic DNA was extracted from dried blood spot samples using a mini-nucleic acid extraction kit (Concert Bio, Xiamen, China), strictly following the manufacturer’s protocol. LRS-based CAH genotyping was performed at Berry Genomics Corporation (Beijing, China), as previously reported (Liu et al., 2022). Briefly, six locus-specific primer pairs were synthesized to generate 7.0–8.5 kb amplicons covering *CYP21A2*, *CYP21A1P*, *CYP11B1*, *CYP17A1*, *HSD3B2*, and *StAR* via long-range PCR. A targeted long-range PCR (LR-PCR) assay was further established for full-length amplification of the functional *CYP21A2-TNXB* haplotype, which enables discrimination against its homologous pseudogene counterpart *CYP21A1P-TNXA*. Two unique terminal primer pairs were designed against non-homologous flanking sequences of the *CYP21A2* promoter and *TNXB* to prevent off-target cross-amplification of *CYP21A1P*. The primer sequences used for full-length capture of the 30.8 kb *CYP21A2* target amplicon were as follows: Forward Long-F: 5′-CAGTCTCCATGTCSCAAAACACGTTC-3′, Reverse Long-R: 5′-GATGGTGGCATTGAGCAAGGGGCAG-3′. An additional six sets of short LR-PCR primers were designed to cover *CYP11B1*, *CYP17A1*, *HSD3B2*, *StAR*, and the chimeric junction regions of *CYP21A2*/*CYP21A1P*. The design strategy of these primers circumvents cross-reactivity between the 96%–98% homologous exon–intron sequences of *CYP21A2* and *CYP21A1P*. All primers anneal to divergent non-homologous segments between the functional gene and its paralogous pseudogene, ensuring exclusive capture of genuine *CYP21A2* alleles for downstream CCS analysis. Subsequently, PCR products were purified, end-repaired, and ligated with unique barcoded adaptors to construct single-molecule real-time (SMRT) bell libraries using the Sequel Binding Kit 2.0 and Internal Control Kit 1.0 (Pacific Biosciences, California, United States). The primed DNA-polymerase complexes were then subjected to SMRT cells and sequenced on the Sequel II platform (Pacific Biosciences, California, United States).

Debarcoded and filtered CCS reads were obtained from the Sequel II platform using CCS software and Lima (included in the Pbbioconda package; Pacific Biosciences, California, United States). These CCS reads were then mapped to the human GRCh38/hg38 reference genome. FreeBayes 1.3.4 was used to identify SNVs and indels. For the analysis of *CYP21A2*, *CYP21A1P*, as well as *CYP21A1P*/*CYP21A2* and *CYP21A2*/*CYP21A1P* chimeric genes, two custom-constructed reference sequences (*CYP21A2*-TNXB and *CYP21A1P*-TNXA) were employed for identification. All variants were classified using two standardized systems: (1) the 2015 ACMG/AMP variant classification guidelines (PMID: 25741868), which stratify variants into pathogenic, likely pathogenic, variant of uncertain significance, likely benign, and benign categories; (2) clinical evidence extracted from the ClinVar public database (https://www.ncbi.nlm.nih.gov/clinvar/) for cross-validation of pathogenic evidence for each annotated variant. Variants detected by the LRS-based genotyping assay that had not been previously reported were validated by Sanger sequencing.

For heterozygous carriers summarized in Tables 1, 2, the phenotypic annotations (SW, SV, NC) assigned to each CYP21A2 variant indicate the disease subtype observed in biallelic affected individuals carrying the same pathogenic allele. Such labels solely reflect the intrinsic residual 21-hydroxylase activity and pathogenic potency of each individual variant, and do not correspond to any clinical phenotype in heterozygous asymptomatic neonates. Subtype categorization of all detected CYP21A2 variants was implemented in accordance with the Endocrine Society clinical practice guideline (Speiser et al., 2018) and the EMQN consensus recommendations for molecular testing of 21-hydroxylase deficiency (Baumgartner-Parzer et al., 2020). Variant pathogenicity and subtype assignments were further cross-validated against the ClinVar public variant database and published genotype–phenotype correlation data from Chinese CAH cohorts (Liang et al., 2025). Consistent with the autosomal recessive inheritance pattern of CAH, heterozygous individuals carrying only one pathogenic allele retain normal adrenal steroidogenesis and remain clinically unaffected.

**TABLE 1:** Variants identified in 52 suspicious-positive CAH newborns.

Gene	Variant type	Nucleotide variation	Phenotype	Carrier allele	Allele number	Frequency % (n = 20)
*CYP21A2*	SNV/Indel	c.293-13C>G	SW/SV	Y	4	20 (4/20)
​	​	c.293-39_293-38delinsGG	SW/SV	Y	3	15 (3/20)
​	​	c.371C>T	NC	Y	2	10 (2/20)
​	​	c.1280G>A	SW	Y	1	5 (1/20)
​	​	c.45C>T	-	N	1	5 (1/20)
​	Deletion chimera	CYP21A1P/A2-CH-3	SW	Y	1	5 (1/20)
​	​	CYP21A1P/A2-CH-1	SW	Y	1	5 (1/20)
​	Duplication	CYP21A2/A1P	-	N	2	10 (2/20)
​	​	CYP21A2	-	N	1	5 (1/20)
​	Complex mutations	c.[844G>T,923dup, 955C>T]	SW	Y	1	5 (1/20)
​	​	c.[293-13C>G, 332_339del,518T>A, 710TA,713T>A]	SW	Y	1	5 (1/20)
​	​	c.[-126C>T,-113G>A]	NC	Y	1	5 (1/20)
​	​	c.[710T>A,713T>A,719T>A]	SW	Y	1	5 (1/20)
*CYP11B1*	SNV/Indel	c.456C>G	-	-	1	-
​	​	c.1122-20A>G	-	-	1	-
*STAR*	SNV/Indel	c.179-14G>A	-	-	3	-
*HSD3B2*	SNV/Indel	c.308-8G>A	-	-	2	-
​	​	c.13T>A	-	-	2	-

Abbreviation: CAH, congenital adrenal hyperplasia; SNV, single nucleotide variant; Indel, insertion/deletion; SW, salt-wasting; SV, simple virilizing; NC, non-classic; Y, allele carrying pathogenic variants or likely pathogenic variants, designated as a carrier allele; N, allele carrying variants of uncertain significance or benign variants, designated as a non-carrier allele; —, not applicable or not determined.

**TABLE 2:** Carrier frequency of variants of CAH genes in the prospective cohort.

Gene	Nucleotide variation	Phenotype	Carrier allele	Allele frequency % (n = 88)	Carrier rate (n = 88)
*CYP21A2*	c.371C>T	NC	Y	0.524 (22/4200)	1:95 (22/2,100)
	c.293-13C>G	SW/SV	Y	0.190 (8/4200)	1:262.5 (8/2,100)
	c.[-126C>T,-113G>A]	NC	Y	0.143 (6/4200)	1:350 (6/2,100)
	c.844G>T	NC	Y	0.119 (5/4200)	1:420 (5/2,100)
	c.955C>T+TNXB/A-CH-2	-	N	0.095 (4/4200)	1:525 (4/2,100)
	TNXA/B-CH-1	SW	Y	0.095 (4/4200)	1:525 (4/2,100)
	c.-126C>T	NC	Y	0.071 (3/4200)	1:750 (3/2,100)
	c.1069C>T	SW	Y	0.048 (2/4200)	1:1,050 (2/2,100)
	c.188A>T	NC	Y	0.048 (2/4200)	1:1,050 (2/2,100)
	c.913G>A	NC	Y	0.048 (2/4200)	1:1,050 (2/2,100)
	c. [955C>T,1069C>T]	SW	Y	0.048 (2/4200)	1:1,050 (2/2,100)
	CYP21A1P/A2-CH-1	SW	Y	0.048 (2/4200)	1:1,050 (2/2,100)
	c.844G>T+CYP21A2/A1P-CH-7	NC	Y	0.048 (2/4200)	1:1,050 (2/2,100)
	c.-103A>G	NC	Y	0.048 (2/4200)	1:1,050 (2/2,100)
	c.518T>A	SV	Y	0.048 (2/4200)	1:1050 (2/2,100)
	CYP21A2/A1P-CH-8	-	N	0.024 (1/4200)	1:2,100 (1/2,100)
	c.-113G>A	NC	Y	0.024 (1/4200)	1:2,100 (1/2,100)
	c.955C>T	SW	Y	0.024 (1/4200)	1:2,100 (1/2,100)
	c.1450dup	SW	Y	0.024 (1/4200)	1:2,100 (1/2,100)
	TNXA/B-CH-1	SW	Y	0.024 (1/4200)	1:2,100 (1/2,100)
	c.[-126C>T,-113G>A,-110T>C,-103A>G]	NC	Y	0.024 (1/4200)	1:2,100 (1/2,100)
	c.[-126C>T,-113G>A,-110T>C]	NC	Y	0.024 (1/4200)	1:2,100 (1/2,100)
	c.518T>A+CYP21A2/A1P-CH-7	NC	Y	0.024 (1/4200)	1:2,100 (1/2,100)
	c.1306C>T	NC	Y	0.024 (1/4200)	1:2,100 (1/2,100)
	c.[-126C>T,-113G>A,-110T>C,-103A>G,92C>T,293-13C>G,332_339del,518T>A, E6_cluster,923dup]	SW	Y	0.024 (1/4200)	1:2,100 (1/2,100)
	c.[-113G>A,-110T>C,-103A>G]	NC	Y	0.024 (1/4200)	1:2,100 (1/2,100)
	c. [188A>T,208G>T]	SV	Y	0.024 (1/4200)	1:2,100 (1/2,100)
	c.[-126C>T,-113G>A,518T]>A	SV	Y	0.024 (1/4200)	1:2,100 (1/2,100)
	c.371C>T,c.518T>A	NC	Y	0.024 (1/4200)	1:2,100 (1/2,100)
	c.374G>A	NC	Y	0.024 (1/4200)	1:2,100 (1/2,100)
	c.1070G>A	SV	Y	0.024 (1/4200)	1:2,100 (1/2,100)
	c.[371C>T,1099C>T]	NC	Y	0.024 (1/4200)	1:2,100 (1/2,100)
*CYP17A1*	c.1466delT	-	-	0.024 (1/4200)	1:2,100 (1/2,100)
​	c.1226C>G	-	-	0.024 (1/4200)	1:2,100 (1/2,100)
*STAR*	c.235G>T	-	-	0.024 (1/4200)	1:2,100 (1/2,100)

Abbreviation: CAH, congenital adrenal hyperplasia; NC, non-classic; SW, salt-wasting; SV, simple virilizing; Y, allele carrying pathogenic variants or likely pathogenic variants, designated as a carrier allele; N, allele carrying variants of uncertain significance or benign variants, designated as a non-carrier allele; —, not applicable or not determined.

## 3 Results

### 3.1 LRS of CAH genes in second-tier newborn screening for CAH

A total of 100,145 newborns were recruited from the Fujian Provincial Newborn Screening Center between January 1 and December 31, 2019. All newborns underwent CAH NBS using a 17α-OHP screening assay. A total of 190 cases were identified as screen-positive, with a positive rate of 0.19% (190/100,145; 95% confidence interval [CI], 0.17%–0.21%). Among the screen-positive neonates, 175 cases were successfully recalled, and an additional dried blood spot specimen was collected for re-evaluation. Of these 175 neonates, 20 cases remained positive in the second screening and underwent genetic diagnosis. Ultimately, 5 neonates were definitively diagnosed with CAH, corresponding to an estimated prevalence of 1:20,029.

A total of 52 full-term neonates (gestational age at delivery ≥ 37 weeks and birth weight ≥ 2,500 g) with positive screening results were selected and subjected to LRS-based CAH genotyping. Among the 52 samples analyzed, 5 cases of pathogenic *CYP21A2* mutations were detected, including 3 samples with SNVs on both alleles, 1 sample with a 30-kb deletion on both alleles, and 1 sample with an SNV on one allele and complex SNVs on the other allele; these findings were consistent with the results of genetic diagnosis (Table 3). We additionally identified 10 neonates harboring monoallelic *CYP21A2* variants. Collectively, these 15 individuals (5 patients with biallelic *CYP21A2* variants plus 10 subjects carrying single *CYP21A2* alleles) exhibited 13 distinct *CYP21A2* genotypes, generating a total of 20 *CYP21A2* alleles for frequency estimation (10 alleles derived from subjects with monoallelic variants and 10 alleles from patients with biallelic variants). Furthermore, two neonates carried variants in *CYP11B1*, three carried variants in *StAR*, and four carried variants in *HSD3B2*. Detailed variant information is summarized in Table 1.

**TABLE 3:** LRS-based genotyping results of 5 patients with 21-OHD in second-tier CAH newborn screening.

Patient ID	Sex	Initial 17α-OHP concentration	Allele 1 (LRS)	Allele 2 (LRS)	Final Genotype (MLPA + Sanger)
1	M	349.50	c.293-13C>G	c.293–13C>G	c.293-13C>G/c.293-13C>G
2	M	532.50	CYP21A1P/A2-CH-3	CYP21A1P/A2-CH-1	CYP21A1P/A2-CH-3/CYP21A1P/A2-CH-1
3	F	60.95	c.293-39_293-38delinsGG	c.371C>T	c.293-39_293-38delinsGG/c.371C>T
4	F	101	c.293-13C>G	c.1280G>A	c.293-13C>G/c.1280G>A
5	M	17.7	c.293-13C>G	c. [710T>A,713T>A,719T>A]	c.293-13C>G/c.[710T>A, 713T>A,719T>A]

Abbreviation: LRS, long-read sequencing; CAH, congenital adrenal hyperplasia; 21-OHD, 21-hydroxylase deficiency; 17α-OHP, 17α-hydroxyprogesterone; M, male; F, female.

### 3.2 LRS of CAH genes in first-tier newborn screening for CAH

Based on the excellent performance of LRS in CAH genetic detection for second-tier NBS, we further explored its clinical feasibility as a first-tier screening strategy. A total of 2,100 newborns (1,123 males and 977 females) underwent parallel detection of serum 17α-OHP and LRS-based CAH genotyping. Two newborns with normal 17α-OHP biochemical screening results were found to carry biallelic pathogenic variants in the *CYP21A2* gene, yielding a prevalence of 1 in 1050 (Table 4). Furthermore, heterozygous variants were detected in 88 neonates (4.2%) exhibiting unremarkable 17α-OHP levels: 85 individuals harbored variants in *CYP21A2*, 2 carried variants in *CYP17A1*, and 1 bore a variant in *StAR*. Thirty-two distinct genotypes were observed among the 85 subjects with *CYP21A2* heterozygous alterations (Table 2). The overall carrier rates were 1:78 (27/2,100) for classic CAH (CCAH) and 1:40 (53/2,100) for non-classic CAH (NCCAH). The allelic variant c.371C>T was the most prevalent among NCCAH carriers, with an allelic frequency of 0.524% (22/4200). Among the 27 carriers with classic phenotype-associated genotypes, c.293-13C>G was the dominant allele, with an allelic frequency of 0.190% (8/4200).

**TABLE 4:** Positive cases identified via LRS-based *CYP21A2* genotyping.

Case ID	Sex	Initial 17α-OHP concentration	Allele 1 genotype	Allele 2 genotype
1	F	1.8	c.[-126C>T,-113G>A]	c.844G>T
2	M	3.1	c.844G>T	c.913G>A

Abbreviation: LRS, long-read sequencing; CAH, congenital adrenal hyperplasia; 17α-OHP, 17α-hydroxyprogesterone; M, male; F, female.

## 4 Discussion

Classic CAH remains a rare disorder, with its prevalence ranging from 1 in 10,000 to 1 in 20,000 across most human populations. In contrast, non-classic CAH occurs far more frequently, with a prevalence ranging from 1 in 200 to 1 in 2,000. Non-classic (mild) CAH is caused by *CYP21A2* variants that preserve 20%–50% of residual enzymatic activity. This subtype is often clinically asymptomatic and highly prevalent. Epidemiological data have revealed that the prevalence of non-classic CAH reaches approximately 1 in 200 in the United States, and its carrier frequency ranges from 4.0% to 7.5% in several European countries (Baumgartner-Parzer et al., 2005; Ezquieta et al., 2005; Phedonos et al., 2013; Hannah-Shmouni et al., 2017).

Neonatal screening for 21-OHD has been routinely performed in Fujian Province, China, for nearly 3 years. The widespread implementation of such newborn screening programs is critical for preventing neonatal adrenal crisis and reducing early mortality. Nevertheless, conventional biochemical screening fails to identify patients with non-classic CAH when their serum 17α-OHP levels stay within the normal reference range. Females affected by non-classic CAH tend to develop hyperandrogenism and infertility in adulthood, whereas most male patients with this subtype remain undiagnosed throughout their lives. Furthermore, given the relatively high false-positive rate of traditional biochemical assays, molecular genetic screening has emerged as an indispensable tool for accurate subtype classification and early definitive diagnosis of CAH.

Single-molecule long-read sequencing has become a mainstream sequencing technology. It enables real-time sequencing at the single-DNA-molecule level and generates substantially longer reads with higher sequencing efficiency (Hu et al., 2021). Owing to its robust detection capability, this technology has been widely applied in the diagnosis of cancers and human genetic disorder (Thibodeau et al., 2020; Xu et al., 2020; Chen et al., 2023; Mimosa et al., 2023). Compared with MLPA combined with Sanger sequencing, which presents obvious drawbacks in clinical performance and economic cost, LRS-based genotyping serves as a promising strategy for CAH newborn screening (Chen et al., 2012; Xu et al., 2013; Concolino, 2019). Firstly, it yields comprehensive genetic profiles, enabling direct analysis of copy number variations and cis-trans configurations of *CYP21A2* and *CYP21A1P*, as well as precise identification of chimeric gene junction site (Liu et al., 2022; Li et al., 2023). Secondly, MLPA and Sanger sequencing suffer from low throughput and demand high-quality genomic DNA at concentrations above 10 ng/μL. By contrast, LRS-based CAH genotyping only requires DNA concentrations exceeding 1 ng/μL and is compatible with neonatal dried blood spot specimens, which better adapts to large-scale newborn genetic screening. Thirdly, LRS achieves faster genotyping turnaround than MLPA, shortening the interval from initial screening to final diagnosis and cutting overall costs in population-scale newborn screening (Liu et al., 2022; Adachi et al., 2024). Furthermore, integrating genetic testing into routine 17α-OHP biochemical screening and conducting integrated result analysis can markedly reduce unnecessary recall procedures and repeated dried blood spot sampling. Meanwhile, non-classic CAH and carrier status can be directly determined, facilitating timely clinical monitoring, early intervention and prenatal genetic counseling for subsequent pregnancies.

In the present study, the estimated CAH prevalence was 1:20029, which is comparable to the national incidence of 1:23024 reported in China. The c.293-13C>G variant was the most prevalent mutation, accounting for 20% of all detected variants, in line with earlier findings (Hou et al., 2019). Within our study, *CYP21A2/A1P* duplication chimeras were categorized as benign variants in accordance with published LRS data for CAH (Liang et al., 2025), for the following reasons: These trimodular RCCX rearrangements harbor two intact wild-type copies of *CYP21A2* without deleterious pseudogene-derived fusion breakpoints that abrogate enzymatic function; no loss-of-function sequences originating from *CYP21A1P* are incorporated into the duplicated functional gene, and *in vitro* enzymatic activity assays confirmed residual 21-hydroxylase activity exceeding 80%. Population genetic data revealed a high carrier frequency of pure duplication chimeras at 4.00%. Furthermore, long-read-based haplotype phasing verified that duplication chimeras only act as pathogenic alleles when they reside in cis with SNVs or indels. This retrospective analysis adopted LRS-based genotyping to establish a combined biochemical and genetic screening model, verifying its clinical value as a second-tier newborn screening approach. Of the 52 neonates who underwent LRS-based CAH genetic testing, no false-positive results were detected except for five individuals with confirmed CAH. Among the 175 newborns recalled due to elevated initial 17α-OHP screening values, only five were finally diagnosed with CAH, demonstrating that approximately 97.1% (170/175) of recall visits were clinically unwarranted. These observations indicate that the current 17α-OHP threshold generates abundant false-positive signals and triggers redundant clinical follow-up. Incorporating LRS-mediated CAH gene analysis as a second-tier newborn screening strategy could effectively minimize unnecessary recall procedures.

Globally, studies focusing on *CYP21A2* mutation carrier screening remain limited. Here, we applied LRS-based CAH genotyping in primary newborn screening to investigate the carrier frequency of *CYP21A2* mutations in Fujian region.

The primary objective of newborn screening is to avert neonatal adrenal crisis and mortality. This is especially critical for male infants, as 21-hydroxylase deficiency (21-OHD) rarely presents with abnormal external genitalia and thus tends to evade clinical detection (Nordenström et al., 2005; Gidlöf et al., 2014). Existing screening strategies generally achieve high sensitivity for identifying salt-wasting 21-OHD (Gidlöf et al., 2014). Serum 17α-OHP levels rise within the first weeks after birth and correlate closely with disease severity and *CYP21A2* genotypes. Early diagnosis of milder subtypes including simple virilizing CAH helps curb premature androgen overexpression, supporting normal physical growth and development. Non-classic 21-OHD is commonly missed by conventional biochemical screening. Given the inevitable false-positive and false-negative results of biochemical assays, molecular genetic testing plays a vital role in CAH subtype classification and clinical management (Wang et al., 2021; Liu et al., 2022).

In this study, 2,100 newborns received simultaneous 17α-OHP detection and LRS-based CAH genotyping. Two neonates presenting normal 17α-OHP levels harbored biallelic pathogenic variants in the *CYP21A2* gene, corresponding to a population prevalence of 1 in 1050. An additional 88 infants (4.2%) carried heterozygous alleles linked to CAH pathogenesis: 85 individuals harbored variants in *CYP21A2*, 2 bore variants in *CYP17A1*, and 1 carried a variant in *StAR*. LRS into first-tier CAH newborn screening enhances diagnostic sensitivity and specificity for NCCAH and yields robust molecular evidence to support genetic counselling. This advantage is particularly notable for families carrying alleles associated with classic CAH, who face elevated disease recurrence risks in future pregnancies. LRS broadens the spectrum of detectable disease-causing variants and enables identification of pathogenic alterations in CAH-related genes that cannot be captured by routine biochemical screening, including *CYP11B1*, *CYP17A1*, *StAR*, and *HSD3B2*. Earlier research focusing on Chinese CAH populations has indicated divergent phenotypic manifestations in individuals carrying biallelic *CYP21A2* variants, even among subjects harboring identical pathogenic alleles. Such variability may stem from modifier gene effects, gestational hormonal milieu, and postnatal stress conditions. Nonetheless, allele-specific phenotypic stratification (SW/SV/NC), which is established on residual enzyme activity and large clinical population datasets, is the primary framework adopted to assess variant pathogenic potential in newborn genetic screening. Nevertheless, this stratification framework cannot be utilized to confirm the clinical CAH status of infants with heterozygous gene alterations.

The combined screening strategy integrating LRS and biochemical testing established in the present study yields an overall pipeline TAT of merely seven calendar days. By contrast, the conventional two-stage workflow involving initial biochemical screening, clinical recall, and subsequent MLPA or Sanger sequencing verification requires a total TAT of 26 calendar days (Wang et al., 2025). The 19-day reduction in diagnostic waiting window eliminates the critical risk window for neonatal adrenal decompensation in SW-CAH patients. In the national 21,239 newborn LRS first-tier screening cohort, only 0.08% of DBS samples required repeat sequencing due to poor DNA quality, confirming stable high-throughput laboratory throughput compatible with routine provincial newborn screening centers (Liang et al., 2025).

LRS genotyping covering the complete CAH gene panel (*CYP21A2*, *CYP11B1*, *CYP17A1*, *HSD3B2*, and *StAR*) costs roughly 20 USD per sample via batch sequencing on barcoded 384-plex PacBio Sequel II platforms. In comparison, single MLPA analysis costs around 16.7 USD per specimen. For samples yielding equivocal MLPA signals, mandatory confirmatory Sanger sequencing incurs an extra 50.1 USD per case; collectively, the combined MLPA and Sanger workflow costs nearly 67 USD for each newborn with an initially suspicious biochemical screening result (Wang et al., 2025). LRS can resolve genotype ambiguity without parental trio validation, removing the financial expenditure incurred by family-based genetic testing. Conventional biochemical screening yields abundant false-positive results, which necessitate repeat heel-prick DBS collection, secondary 17α-OHP testing, and stepwise MLPA plus Sanger molecular validation. Every false-positive infant brought back for clinical follow-up accumulates extra expenditures including nursing sampling manpower, cold-chain logistics, repeated biochemical reagents, and multiple rounds of molecular testing. As a first-tier screening modality, LRS substantially cuts expenditures from unneeded patient recalls and duplicate biochemical assays. Overall, adopting LRS as primary screening reduces auxiliary recall-associated costs by an estimated 62% among all infants with abnormal initial screening readouts (Wang et al., 2025).

This study identified 19 neonates harboring single heterozygous CAH alleles from the retrospective cohort of 52 screen-positive infants, and an additional 88 individuals with heterozygous CAH variants among the 2,100 prospective newborns who exhibited normal 17α-OHP levels. While data on heterozygous variant prevalence yields valuable population genetic evidence, universal NBS is principally designed to detect infants with biallelic pathogenic variants who require urgent early clinical intervention. This creates ethical concerns over routinely disclosing results indicating single heterozygous variants to parents. Nevertheless, such genetic findings carry meaningful value for families’ reproductive planning. If both parents carry a heterozygous pathogenic CYP21A2 allele, each subsequent pregnancy carries a 25% risk of offspring affected by classic CAH. Early detection of heterozygous variants in newborns allows timely genetic testing for parents and targeted prenatal diagnosis in future pregnancies, lowering the risk of recurrent affected infants. This advantage is particularly notable in populations with higher frequencies of pathogenic classic CAH alleles. Clinical screening centers should establish standardized genetic counseling workflows for carrier results. Dedicated genetic counselors provide separate, tailored interpretation of heterozygous variants to parents, clearly differentiating asymptomatic carrier status from clinically affected individuals with biallelic pathogenic variants, thereby mitigating undue parental anxiety stemming from ambiguous carrier reports.

This study has several key limitations. First, its single-center design restricts the external generalizability of our findings. Large multicenter prospective studies are thus needed to characterize the epidemiological profile of CAH in Fujian Province, verify the clinical performance of LRS-combined screening, and systematically evaluate the cost-effectiveness of integrated biochemical and genetic newborn screening algorithms. Second, the two genetically identified NCCAH neonates in the prospective cohort lacked confirmatory ACTH stimulation tests and adrenal androgen measurements in the neonatal period. Long-term outpatient follow-up through adolescence has been arranged, which will facilitate definitive phenotypic validation. The lack of neonatal functional assays creates mild diagnostic ambiguity for these two subjects. Third, we did not develop a comprehensive health economic model incorporating multi-dimensional expenditure indicators. Further multicenter prospective trials are warranted to calculate precise population-level cost-effectiveness ratios.

## 5 Conclusion

In conclusion, this study verifies that integrating LRS-based genetic testing with conventional biochemical detection serves as an optimized and effective newborn screening strategy for CAH. This combined model markedly lowers false-positive and recall rates, relieves parental concerns, and substantially elevates overall screening efficiency. Meanwhile, this work confirms the clinical performance of targeted LRS genetic screening, which acts as a valuable supplementary tool for primary CAH screening. It effectively decreases false-positive and false-negative results, accelerates diagnostic procedures, and enables simultaneous identification of affected patients and gene carriers. Although our findings support the promising application prospect of LRS in CAH newborn screening, large-sample multicenter prospective trials are still needed to fully assess its clinical practicability and economic benefits in population-scale screening. Such evidence will facilitate the widespread clinical adoption and popularization of this screening modality.

## Data Availability

The datasets presented in this study can be found in online repositories. The names of the repository/repositories and accession number(s) can be found in the article/supplementary material.

## References

[B1] Adachi E. Nakagawa R. Tsuji-Hosokawa A. Gau M. Kirino S. Yogi A. (2024). A MinION-based long-read sequencing application with one-step PCR for the genetic diagnosis of 21-Hydroxylase deficiency. J. Clin. Endocrinol. Metab. 109, 750–760. 10.1210/clinem/dgad577 37804107

[B2] Auer M. K. Nordenström A. Lajic S. Reisch N. (2023). Congenital adrenal hyperplasia. Lancet 401, 227–244. 10.1016/S0140-6736(22)01330-7 36502822

[B3] Baumgartner-Parzer S. M. Nowotny P. Heinze G. Waldhäusl W. Vierhapper H. (2005). Carrier frequency of congenital adrenal hyperplasia (21-Hydroxylase deficiency) in a Middle European population. J. Clin. Endocrinol. Metab. 90, 775–778. 10.1210/jc.2004-1728 15572419

[B4] Baumgartner-Parzer S. Witsch-Baumgartner M. Hoeppner W. (2020). EMQN best practice guidelines for molecular genetic testing and reporting of 21-hydroxylase deficiency. Eur. J. Hum. Genet. 28, 1341–1367. 10.1038/s41431-020-0653-5 32616876 PMC7609334

[B5] Carvalho B. Marques C. J. Santos-Silva R. Fontoura M. Carvalho D. Carvalho F. (2021). Congenital adrenal hyperplasia due to 21-Hydroxylase deficiency: an update on genetic analysis of CYP21A2 gene. Exp. Clin. Endocrinol. and Diabetes 129, 477–481. 10.1055/a-1108-1419 32131114

[B6] Chen W. Xu Z. Sullivan A. Finkielstain G. P. Van Ryzin C. Merke D. P. (2012). Junction site analysis of chimeric CYP21A1P/CYP21A2 genes in 21-Hydroxylase deficiency. Clin. Chem. 58, 421–430. 10.1373/clinchem.2011.174037 22156666 PMC5576027

[B7] Chen X. Harting J. Farrow E. Thiffault I. Kasperaviciute D. Hoischen A. (2023). Comprehensive SMN1 and SMN2 profiling for spinal muscular atrophy analysis using long-read PacBio HiFi sequencing. Am. J. Hum. Genet. 110, 240–250. 10.1016/j.ajhg.2023.01.001 36669496 PMC9943720

[B8] Concolino P. (2019). Issues with the detection of large genomic rearrangements in molecular diagnosis of 21-Hydroxylase deficiency. Mol. Diagn. Ther. 23, 563–567. 10.1007/s40291-019-00415-z 31317337

[B9] El-Maouche D. Arlt W. Merke D. P. (2017). Congenital adrenal hyperplasia. Lancet 390, 2194–2210. 10.1016/S0140-6736(17)31431-9 28576284

[B10] Ezquieta B. Ruano M. L. F. Dulín E. Arnao D. R. Rodríguez A. (2005). Prevalencia de enfermedades recesivas frecuentes en población española mediante análisis de ADN en muestras del cribado neonatal. Med. Clin. Barc. 125, 493–495. 10.1157/13080213 16238926

[B11] Falhammar H. Nordenström A. (2015). Nonclassic congenital adrenal hyperplasia due to 21-hydroxylase deficiency: clinical presentation, diagnosis, treatment, and outcome. Endocrine 50, 32–50. 10.1007/s12020-015-0656-0 26082286

[B12] Gidlöf S. Wedell A. Guthenberg C. von Döbeln U. Nordenström A. (2014). Nationwide neonatal screening for congenital adrenal hyperplasia in Sweden. JAMA Pediatr. 168, 567. 10.1001/jamapediatrics.2013.5321 24733564

[B13] Hannah-Shmouni F. Morissette R. Sinaii N. Elman M. Prezant T. R. Chen W. (2017). Revisiting the prevalence of nonclassic congenital adrenal hyperplasia in US Ashkenazi jews and Caucasians. Genet. Med. 19, 1276–1279. 10.1038/gim.2017.46 28541281 PMC5675788

[B14] Hayashi G. Y. Carvalho D. F. de Miranda M. C. Faure C. Vallejos C. Brito V. N. (2017). Neonatal 17 hydroxyprogesterone levels adjusted according to age at sample collection and birthweight improve the efficacy of congenital adrenal hyperplasia newborn screening. Clin. Endocrinol. (Oxf). 86, 480–487. 10.1111/cen.13292 27978607

[B15] Hou L. Liang L. Lin S. Ou H. Liu Z. Huang S. (2019). Analysis of phenotypes and genotypes in 84 patients with 21-Hydroxylase deficiency in southern China. Steroids 151, 108474. 10.1016/j.steroids.2019.108474 31446012

[B16] Hu T. Chitnis N. Monos D. Dinh A. (2021). Next-generation sequencing technologies: an overview. Hum. Immunol. 82, 801–811. 10.1016/j.humimm.2021.02.012 33745759

[B17] Li H. Zhu X. Yang Y. Wang W. Mao A. Li J. (2023). Long-read sequencing: an effective method for genetic analysis of CYP21A2 variation in congenital adrenal hyperplasia. Clin. Chim. Acta 547, 117419. 10.1016/j.cca.2023.117419 37276943

[B18] Liang D. Zhu M. Liang Q. Qiang R. Yu L. Xu S. (2025). Genetic characterization and screening of congenital adrenal hyperplasia by long-read sequencing in a cohort of 21,239 newborns. Genome Med. 18, 15. 10.1186/s13073-025-01594-7 41469707 PMC12866592

[B19] Liu Y. Chen M. Liu J. Mao A. Teng Y. Yan H. (2022). Comprehensive analysis of congenital adrenal hyperplasia using long-read sequencing. Clin. Chem. 68, 927–939. 10.1093/clinchem/hvac046 35714169

[B20] Merke D. P. Auchus R. J. (2020). Congenital adrenal hyperplasia due to 21-Hydroxylase deficiency. N. Engl. J. Med. 383, 1248–1261. 10.1056/NEJMra1909786 32966723

[B21] Miller W. L. (2018). Mechanisms in endocrinology: rare defects in adrenal steroidogenesis. Eur. J. Endocrinol. 179, R125–R141. 10.1530/EJE-18-0279 29880708

[B22] Mimosa M. L. Al-ameri W. Simpson J. T. Nakhla M. Boissinot K. Munoz D. G. (2023). A novel approach to detect IDH point mutations in gliomas using nanopore sequencing. J. Mol. Diagnostics 25, 133–142. 10.1016/j.jmoldx.2022.12.001 36565986

[B23] Nordenström A. Ahmed S. Jones J. Coleman M. Price D. A. Clayton P. E. (2005). Female preponderance in congenital adrenal hyperplasia due to CYP21 deficiency in England: implications for neonatal screening. Horm. Res. Paediatr. 63, 22–28. 10.1159/000082896 15627780

[B24] Phedonos A. Shammas C. Skordis N. Kyriakides T. Neocleous V. Phylactou L. (2013). High carrier frequency of 21‐hydroxylase deficiency in Cyprus. Clin. Genet. 84, 585–588. 10.1111/cge.12153 23600966

[B25] Saraf S. Srivastava P. Panigrahi I. Seenappa V. Kumar R. Yadav J. (2024). Characterization of the CYP21A2 gene mutations in children with classic congenital adrenal hyperplasia. Indian J. Pediatr. 91, 137–142. 10.1007/s12098-021-03975-3 35094236

[B26] Speiser P. W. Arlt W. Auchus R. J. Baskin L. S. Conway G. S. Merke D. P. (2018). Congenital adrenal hyperplasia due to steroid 21-Hydroxylase deficiency: an endocrine society* clinical practice guideline. J. Clin. Endocrinol. Metab. 103, 4043–4088. 10.1210/jc.2018-01865 30272171 PMC6456929

[B27] Thibodeau M. L. O’Neill K. Dixon K. Reisle C. Mungall K. L. Krzywinski M. (2020). Improved structural variant interpretation for hereditary cancer susceptibility using long-read sequencing. Genet. Med. 22, 1892–1897. 10.1038/s41436-020-0880-8 32624572 PMC7605438

[B28] Wang W. Han R. Yang Z. Zheng S. Li H. Wan Z. (2021). Targeted gene panel sequencing for molecular diagnosis of congenital adrenal hyperplasia. J. Steroid Biochem. Mol. Biol. 211, 105899. 10.1016/j.jsbmb.2021.105899 33864926

[B29] Wang X. Lu X. Zheng F. Lin K. Liao M. Dong Y. (2025). Assessment of long-read sequencing-based congenital adrenal hyperplasia genotyping assay for newborns in Fujian, China. Int. J. Neonatal Screen. 11, 22. 10.3390/ijns11010022 40136637 PMC11942758

[B30] Witchel S. F. (2017). Congenital adrenal hyperplasia. J. Pediatr. Adolesc. Gynecol. 30, 520–534. 10.1016/j.jpag.2017.04.001 28450075 PMC5624825

[B31] Xu Z. Chen W. Merke D. P. McDonnell N. B. (2013). Comprehensive mutation analysis of the CYP21A2 gene. J. Mol. Diagnostics 15, 745–753. 10.1016/j.jmoldx.2013.06.001 PMC5803549 24071710

[B32] Xu L. Mao A. Liu H. Gui B. Choy K. W. Huang H. (2020). Long-Molecule sequencing. J. Mol. Diagnostics 22, 1087–1095. 10.1016/j.jmoldx.2020.05.004 32473995

